# New therapeutic modalities in drug discovery and development: Insights & opportunities

**DOI:** 10.5599/admet.1209

**Published:** 2021-12-15

**Authors:** Manfred Kansy, Giulia Caron

**Affiliations:** 1 Independent Consultant, 79111, Freiburg im Breisgau, Germany (manfred.kansy1@gmail.com); 2 Department of Molecular Biotechnology and Health Sciences, University of Torino, Quarello 15, 10135 Torino, Italy (giulia.caron@unito.it)

The study of the statistics of new drug registrations over time shows an interesting pattern. At the end of the last century, productivity, measured in terms of market launches, was declining and charts as the one shown in Figure 1 have often been used to highlight potential productivity losses in pharmaceutical R&D [1,2]. This trend seems to have reversed in the last decade. Certainly, the number of launches alone is not a good measure of productivity and other, partly controversially discussed measures have been applied [3-5].

What might be the reason for this potentially positive trend of increasing numbers of NMEs and Biologics approvals over the last ten years?

It is obvious that massive efforts in strategy adjustments, early ADMET optimization and the introduction of new assays, methods and computational techniques by pharmaceutical R & D and academia have had an effect. New disease-relevant targets have been identified and potential improvements in regulatory processes could have been beneficial. Moreover, the introduction of new therapeutic modalities, besides the so-called classical Rule of 5 (Ro5) [7] compliant molecules, has had some impact.

Antibodies, antibody-drug conjugates, fusion proteins, neurotoxins, peptides, polymers, small interfering RNA, vaccines, etc., are reaching the market. Furthermore, the introduction of the so-called “beyond rule of five compounds” (bRo5) indicates that even for some demanding targets larger compounds (MW >> 500) can be designed and optimized to have adequate ADMET profiles and resulting bioavailability with desired *in vivo* effect. Although general strategies to design oral bioavailable bRo5 drug candidates have not yet been reported, conformational flexibility has been identified as an important parameter to describe the ADME profile of large and flexible derivatives. For example, it has been recently shown that flexibility descriptors helped to improve computational solubility prediction of bRo5 compounds [8]. Moreover, many bRo5 compounds seem to have an inherent potential to conformationally adjust to the surrounding media and behave like molecular chameleons. For instance, the formation of intramolecular hydrogen bonds in an environment-dependent way has been analyzed to explain the passive permeability skills of some new therapeutic modalities [9,10].

Certainly, small molecules still make up the majority of new drug market introductions, and their urgent need in the treatment of newly discovered diseases and the improvement of current therapies is impressively demonstrated during the severe ongoing Covid 19 pandemic.

This special issue of ADMET and DMPK describes new therapeutic modalities, innovative compound characterization tools for drug safety evaluation/optimization and newly developed computational prediction tools for estimating drug brain uptake as examples of how the drug discovery and research fields are continuously innovating and adapting to the changing needs.

In a first impressive work, experts in the field of degraders, i.e., bifunctional compounds that promote degradation of target molecules instead of inhibiting them, also called PROTACs (PROteolysis TArgeting Chimeras), have analyzed degrader data in terms of target distribution [11]. Moreover, they impressively demonstrate how such large and partially flexible bRo5 drug molecules can be engineered in a more rational way to improve ADMET properties. This excellent work by Giuseppe Ermondi and his colleagues at the University of Turin may be of even greater interest, as preliminary results from a recent phase 1 study show that the degrader principle appears to work in humans [12,13].

Colleagues from the Russian Academy of Sciences in Saratov describe how Selenium NanoParticles (SeNPs) can be used to increase the bioavailability of silymarin [14], a drug with discussed liver-protective and anti-cancer activity [15] but unfavorable physicochemical properties. By combining the anti-cancer activity of SeNPs with the effect of poorly soluble silymarin, the in vitro activity could be increased and the side effects of Se could be partially alleviated. This can be considered a good example of how the therapeutic effects and side effects of NP can be potentially controlled by combining drugs in NPs in the future.

In addition to new therapeutic principles, innovative new methods for testing and optimizing drug safety are urgently needed. In a fascinating mini-review [16], a joint team of experts from academia in Australia and the pharmaceutical industry in Switzerland described how 3D bioprinted heart cells could be strategically used in pharmaceutical research and development and potentially improve and streamline regulatory processes.

Finally, a team of experts from the Universities of Naples and Edinburgh describes the development of in silico prediction models based on Immobilized Artificial Membrane (IAM) chromatography measurements and predicted drug properties to estimate the blood-brain barrier passage of drugs [17]. Although the method is premature, due to the currently limited amount of data, it might be applied in drug discovery and development in selecting appropriate drug candidates with improved brain penetration properties in the future.

Overall, this special issue of ADMET and DMPK provides interesting insights into selected innovative areas of drug discovery and development.

## Figures and Tables

**Figure 1.**
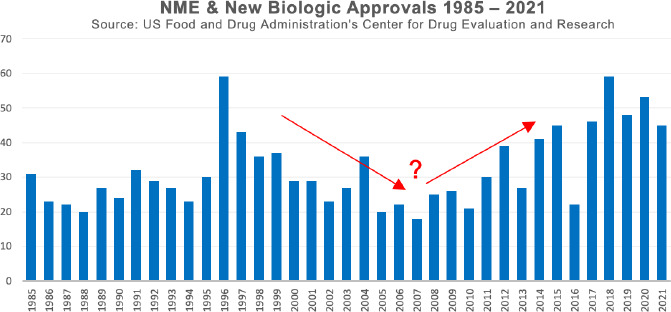
Visualization of CDER New Molecular Entity (NME) Drug and New Biologic approvals 1985-2021 [6].
